# Simulation of infrared spectra of trace impurities in silicon wafers based on the multiple transmission–reflection infrared method

**DOI:** 10.1038/s41598-020-80883-0

**Published:** 2021-01-13

**Authors:** Xiaobin Lu

**Affiliations:** School of Chemistry and Chemical Engineering, Qiannan Normal University for Nationalities, Duyun, 558000 Guizhou China

**Keywords:** Chemistry, Optics and photonics

## Abstract

The content of trace impurities, such as interstitial oxygen and substitutional carbon, in silicon is crucial in determining the mechanical and physical characteristics of silicon wafers. The traditional infrared (IR) method is adopted as a normal means to analyse their concentration at home and abroad, but there are two problems. The first problem is the poor representativeness of a single local sampling point because the impurity distribution in a solid sample is not as uniform as that in a liquid sample. The second problem is that interference fringes appear in the infrared spectra of the sample due to the thin wafer (≤ 300 μm thick). Based on this, controversial issues existed regarding the measured trace impurity concentrations between wafer manufacturers and solar cell assembly businessmen who used silicon sheets made by the former. Therefore, multiple transmission-reflection (MTR) infrared (IR) spectroscopy was proposed to solve the problems mentioned above. In the MTR setup, because light passes through different parts of the silicon chip several times, multiple sampling points make the final result more representative. Moreover, the optical path is lengthened, and the corresponding absorbance is enhanced. In addition to amplification of weak signals, the MTR-IR method can eliminate interference fringes via the integrating sphere effect of its special configuration. The signal-to-noise ratio of the corresponding spectrum is considerably improved due to the aforementioned dual effects. Thus, the accuracy and sensitivity of the detection method for trace impurities in silicon chips are greatly increased. In this study, silicon wafers were placed in the MTR setup, and then, their relative properties at room temperature were investigated. The corresponding theoretical calculation and simulation of infrared spectra of silicon chips were provided. This affords an optional method for the semiconductor material industry to analyse trace impurities in their chips.

## Introduction

With the development of solar cells, the quantity demanded of silicon wafers is increasing. The content of trace impurities in silicon wafers is crucial in determining their physical and mechanical properties. Jiyang Li concluded that a certain concentration of interstitial oxygen in silicon could significantly reduce the effective life of the silicon substrate carriers; therefore, it would lower the efficiency of solar cells^1^. Interstitial oxygen and substitutional carbon in silicon show the greatest influence on the performance of silicon wafers, whereas other types of oxygen and carbon have no effect. Destructive methods, such as gas fusion analysis^2,3^, secondary ion mass spectrometry^4,5^, helium activation analysis^6^, and luminescence activation analysis^7^, are used to analyse the content of oxygen and carbon in silicon wafers. The result obtained is the total element content of oxygen and carbon rather than that of their real specific interstitial and substitutional forms bonded in silicon wafers.

In China, GB/T 32281-2015, test method for measuring oxygen, carbon, boron and phosphorus in solar silicon wafers and feedstock-Secondary ion mass spectrometry, is carried out^8^, but it can destroy silicon chips and obtains the total element content of oxygen and carbon. GB/T 38976-2020, test method for the oxygen concentration in silicon materials-Inert gas fusion infrared detection method, is also performed^9^, but it takes a longer time, can melt silicon chips and obtains the total element content of oxygen and carbon. GB/T 35306-2017, test method for carbon and oxygen content of single crystal silicon-Low temperature Fourier transform infrared spectrometry^10^, can remove some interference fringes; however, it takes a longer time and consumes much power in the process of refrigeration, and its window materials can cause loss of light.

At present, the nondestructive infrared (IR) method, which can keep the chip intact, is commonly used to analyse the content of interstitial oxygen and substitutional carbon in silicon at home and abroad^11–14^. However, the distribution of interstitial oxygen and substitutional carbon in solid silicon samples is heterogeneous, unlike that in liquid solution. In addition, this traditional IR method only has one sampling point for each measurement, so the number of sampling points is insufficient to represent the real condition. Some commercial disputes based on the above reasons arose between silicon chip manufacturers (e.g., Wacker Company, Hemlock Company, OCI Company, and Golden Concord-Zhongneng Silicon Industry Company) and their downstream users, that is, solar cell module manufacturers (e.g., Yingli Company, Trina Solar Company, Hanwha Solarone Company, and Ja Solar Company) who usually used the traditional IR method.

Silicon wafers 100–300 μm thick are often used to fabricate ultrathin solar cells. However, the traditional IR technique cannot measure silicon wafers with a thickness of 100–300 μm, which is less than the wavelength of incident light, thereby resulting in severe interference fringes in infrared spectra. The interference fringes strongly cover the absorption peaks.

**Figure 1:**
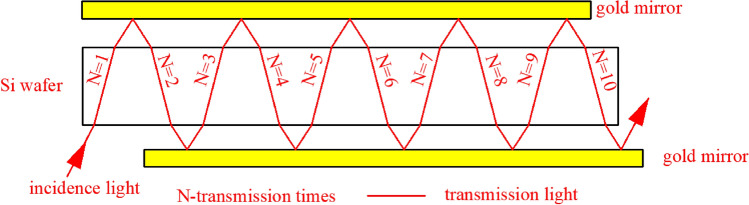
Schematic of the MTR equipment. The incident light undergoes multiple transmissions through and reflections on a silicon wafer placed between two gold mirrors. For clarity and simplicity, the transmitted light is shown without the reflected part. The red line indicates that the IR beam passes through the silicon chip *N* = 10 times. The maximum *N* can reach 14 by extending the length of the gold mirrors and silicon wafer. The image was created using AutoCAD 2014 (simplified Chinese), http://win.xmjfg.com/pg/246.html.

To solve these problems, multiple transmission-reflection (MTR) infrared (IR) spectroscopy was proposed. As shown in Fig. 1, incident light enters and is transmitted through a silicon chip and re-enters it via reflection from golden mirrors placed on both sides of the silicon slice.

The first advantage of the MTR-IR method is that the number of sampling points can be increased to a dozen times that in the traditional IR method, so the final result is more representative. Second, this MTR-IR method can eliminate interference fringes in spectra caused by thin wafers, making it suitable for analysing the thinner wafers (≤ 300 μm thick) often used by all solar cell module manufacturers, such as Yingli Company, Trina Solar Company, Hanwha Solarone Company, and Ja Solar Company. Third, the MTR-IR method can increase the optical path ten times; thereby, the same enhancement occurs for the absorbance signal. Thus, the MTR-IR technique can decrease the limit of detection of impurities in silicon wafers with the same thickness by an order of magnitude compared with the traditional IR method. Fourth, due to the integrating sphere effect generated by the special MTR-IR structure, when transmitting light and reflecting light with the same phase meet, their wave crests and troughs are added and offset, which can eliminate interference fringes in spectra. Therefore, the signal-to-noise ratio of the infrared spectra can be greatly improved by this method.

Professor Xiao’s group successfully applied MTR-IR technology in qualitative and quantitative measurements of ultrathin organic, macromolecular, and biological molecules on silicon surfaces at room temperature, in which the absorbance ratio of s- to p-polarized light that they obtained could be used to calculate the monolayer molecule group orientation on the surface relative to the substrate^15–19^. Several teams abroad used the MTR-IR method for scientific research in various fields. Prof. Spencer investigated the tribological and other characteristics of organic molecules and polymer brushes on the surface of silicon wafers by using the MTR-IR method as the main tool^20,21^. He also studied the composition and orientation of various functional molecules fabricated by different film-making methods^22,23^. Application of the MTR-IR method in the determination of molecular orientation can also be used to explore the effect of chromophore orientation on the photoelectric conversion efficiency of solar cells and organic light-emitting diode (OLED) devices.

MTR-IR technology has a bright future. In view of the manpower and financial resources, this study mainly focused on MTR-IR analysis experiments and theoretical simulations of infrared spectra of silicon wafers.

## Experimental

### Silicon wafer data

Double-side-polished silicon wafers (<100>-oriented, n-type, B-doped, 2000 (CZ) and 8000 (FZ) Ω cm resistivity, and 200 μm, 300 μm and 500 μm thick from Fujian Hanchen New Materials Company Limited, China) were cut into rectangular shapes (15 mm × 50 mm) for infrared analysis. CZ silicon wafers were used as samples and FZ as references to analyse the content of interstitial oxygen and substitutional carbon in silicon.

### Silicon wafer cleaning

Silicon wafers were washed with a mixture of 98% H_2_SO_4_/30% H_2_O_2_ (3:1, V/V) at boiling point for 4 h to remove organic pollutants, followed by boiling in a solution of NH_3_.H_2_O/H_2_O/H_2_O_2_ (1:1:1, V:V:V) for 30 min, cooling to room temperature, rinsing with deionized water, and storage in deionized water. Silicon chips were immersed in 1% HF for 5 min to remove the native silicon oxide layer and dried using N_2_ gas before infrared analysis.

### Analysis

The optical equipment was designed for adaption to any commercial FTIR spectrometer, which was a Bruker Tensor 27 in our case. A Brewster incidence angle of 74°, a deuterium triglyceride sulfate detector and scan times of 100 at 4 cm^−1^ resolution were used for analysis over the middle infrared wavenumber range from 400 to 4000 cm^−1^ in the MTR-IR method.

The entire analysis procedure was carried out according to SEMI MF 1188-2007 and SEMI MF 1391–2007^13,14,24,25^. Each silicon sample was analysed successively four times by the MTR-IR and traditional IR methods by slightly relocating the Si wafer each time to analyse different sampling points. Therefore, for each silicon sample, four different sampling points were analysed by the traditional IR method, whereas 40 different sampling points were analysed by the MTR-IR method with N = 10.

The simulation programme in this article was written using Matlab R2017a (simplified Chinese, trials), https://ww2.mathworks.cn/campaigns/products/trials.html.

## Simulation theory

### Interference elimination function of an integrating sphere^26^

The MATLAB program for calculation of an integrating sphere can be seen in the supporting information. When original light passes through a silicon sheet once, the attenuation factor U (Eq. (2)) of light caused by silicon wafer absorption, reflectance R (Eq. (3)), and transmittance T (Eq. (4)) are given in the following formulas^26^

1 $$\delta^{^{\prime}} = 2\pi \sigma bN_{z}$$

2 $$U = \exp ( - 2\pi \sigma bK)$$

3 $$R = \frac{{\left| {r_{1} } \right|^{2} + U^{4} \left| {r_{2} } \right|^{2} + 2U^{2} \left| {r_{1} } \right|\left| {r_{2} } \right|\cos (2\delta^{^{\prime}} + \phi_{1r} - \phi_{2r} )}}{{1 + U^{4} \left| {r_{1} } \right|^{2} \left| {r_{2} } \right|^{2} + 2U^{2} \left| {r_{1} } \right|\left| {r_{2} } \right|\cos (2\delta^{^{\prime}} - \phi_{1r} - \phi_{2r} )}}$$

4 $$T = \frac{{n_{2} \cos \theta_{2} }}{{n_{1} \cos \theta_{1} }}\frac{{U^{2} \left| {t_{1} } \right|^{2} \left| {t_{2} } \right|^{2} }}{{1 + U^{4} \left| {r_{1} } \right|^{2} \left| {r_{2} } \right|^{2} + 2U^{2} \left| {r_{1} } \right|\left| {r_{2} } \right|\cos (2\delta^{^{\prime}} - \phi_{1r} - \phi_{2r} )}}$$

5 $$2N_{z}^{2} = n^{2} - \kappa^{2} - n_{1}^{2} \sin \theta_{1}^{2} + \sqrt {(n^{2} - \kappa^{2} - n_{1}^{2} \sin \theta_{1}^{2} )^{2} + 4n^{2} \kappa^{2} }$$

6 $$2K^{2} = - (n^{2} - \kappa^{2} - n_{1}^{2} \sin \theta_{1}^{2} ) + \sqrt {(n^{2} - \kappa^{2} - n_{1}^{2} \sin \theta_{1}^{2} )^{2} + 4n^{2} \kappa^{2} }$$

7 $$N_{Z} = n * \cos (\theta_{2} ).$$

**Figure 2:**
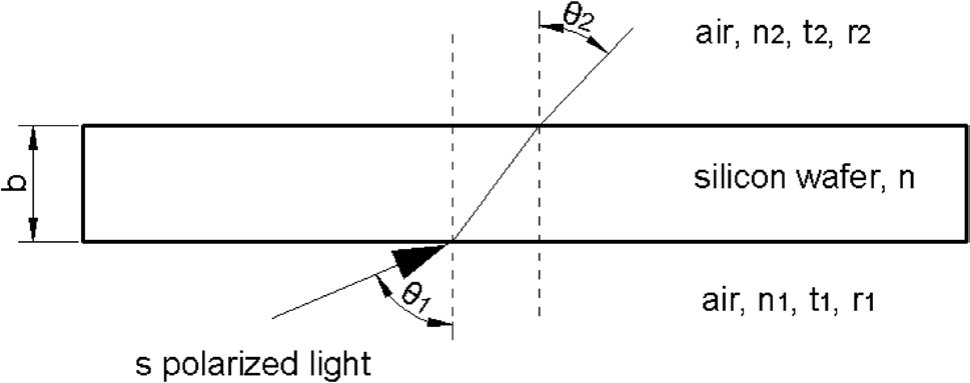
Schematic of reflectance R and transmittance T of s-polarized light transmitting through a thin silicon wafer. The image was created using AutoCAD 2014 (simplified Chinese), http://win.xmjfg.com/pg/246.html.

In Fig. 2, *n* refers to the real part of the complex refractive coefficient of silicon; *κ* denotes its imaginary part; *b* represents the thickness of the silicon wafer, cm; *n*_*1*_ and *n*_2_ correspond to the refractive index of air, with $n_{1} = n_{2}$; *θ*_*1*_ and *θ*_*2*_ specify the incidence angles of light from air to silicon and from silicon to air, respectively; *r*_*1*_ and *r*_*2*_ denote the Fresnel reflection coefficients at the first and second interfaces, respectively; and *t*_*1*_ and *t*_*2*_ refer to the Fresnel transmission coefficients at the first and second interfaces, respectively. *φ*_*1r*_ and *φ*_*2r*_ denote the reflection phase changes at the first and second interfaces, respectively. *N*_*Z*_ indicates the projection of *n* onto the z axis. *K* is the angle between the normal line of the isophase surface of light and z axis. σ is the wavenumber, cm^−1^. *δ’* represents the phase thickness of the silicon wafer. *T* is the transmittance, %; *R* is the reflectance, %; and *U* is the amplitude attenuation factor.

**Figure 3:**
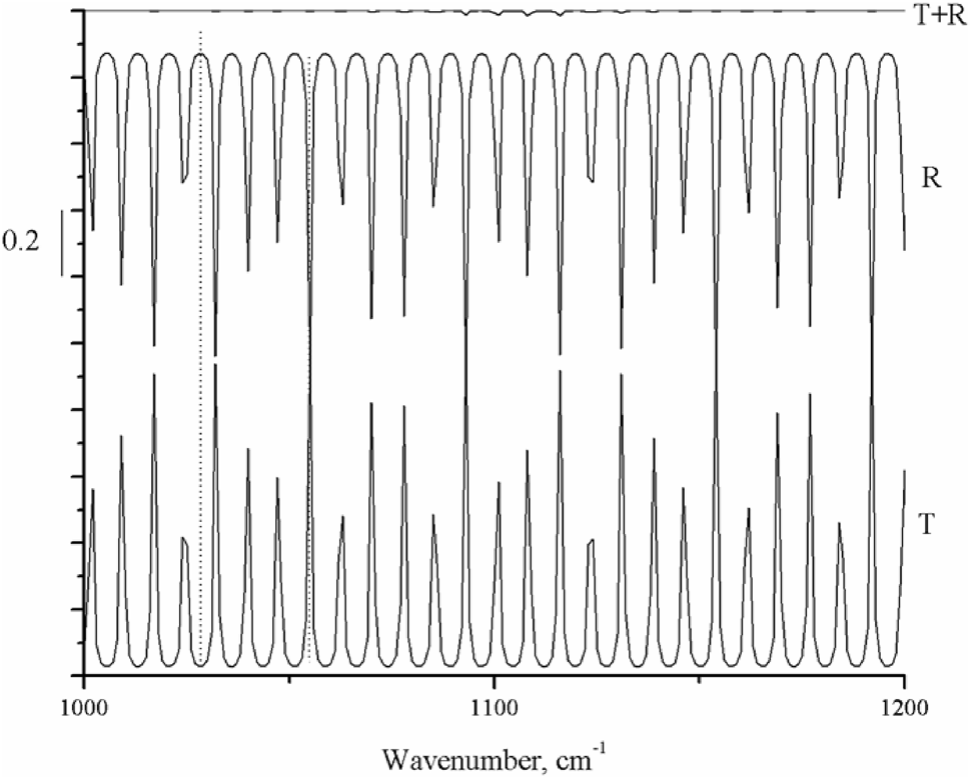
Simulation of reflectance R, transmittance T and addition of R + T of s-polarized light vs. wavenumber. MTR parameters for calculation: incidence angle = 74°, n_1_ = 1.0, and b = 0.02 cm. The image was created using Origin 2017 (simplified Chinese), http://www.32r.com/soft/16769.html.

Figure 3 shows that peaks and troughs periodically appear in the reflection R and transmission T profiles, in which the crest of the R curve corresponds to the trough of T, and vice versa. After the addition of R and T, their crests and troughs cancel out, and a curve that is almost a straight line is obtained. In the MTR setup, when the original light reaches the surface of a silicon chip, it splits into reflected and transmitted parts. The reflected light and transmitted light are reflected by gold mirrors on both sides of the silicon chip and re-enter it. At this point, the wave crest of reflected light correspondingly offsets the trough of transmitted light, and vice versa, thereby eliminating the interference fringes generated by the thin silicon chip. Thus, the quality of the IR spectrum acquired is improved. This phenomenon is the same as reflection light meeting transmission light in an integrating sphere, that is, reducing interference and increasing absorption^27^. However, there is a slight difference between the integrating sphere and the MTR setup. Spherical reflection appears in the former, whereas planar reflection appears in the latter. In addition to the increased optical path length, enlargement of the absorption peak in the MTR-IR spectrum was also achieved due to the energy brought into the sample by the light reflected by gold mirrors. In the MTR-IR method, the interference fringes arising from a thin silicon wafer are eliminated for two reasons: the use of the Brewster incidence angle and the integrating sphere effect of the MTR geometry.

### Simulation and calculation of the infrared spectra of s-polarized light passing through a thin silicon wafer in the MTR-IR method

**Figure 4:**
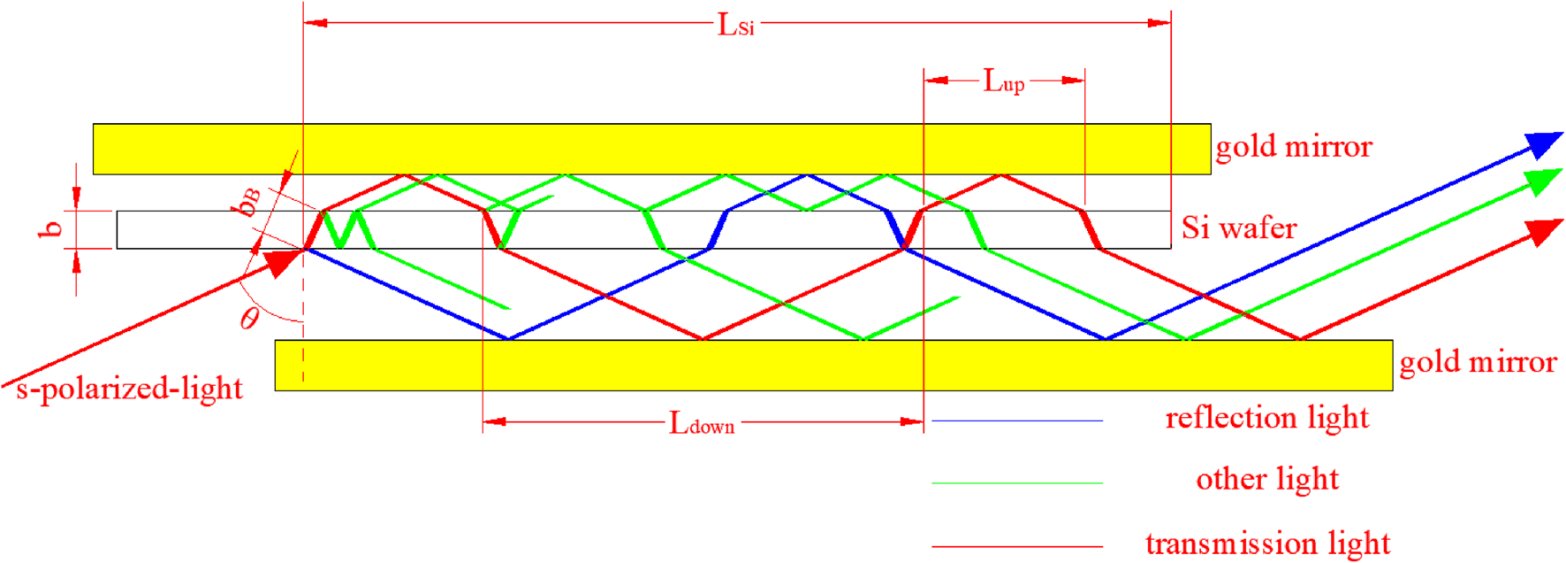
Optical path diagram of s-polarized light in the MTR setup. Only a few transmission times (N = 4) are illustrated for simplicity and clarity. However, in reality, N can reach a maximum of 12. b_B_ corresponds to the optical path length of light passing through the silicon wafer once at the Brewster incidence angle, cm. L_up_ and L_down_ are defined as the periodic horizontal lengths from a spot on the upper and lower faces of silicon where a single ray impinges to the next spot where the ray impinges on the upper and lower surfaces of the silicon wafer again after returning from a gold mirror, respectively. L_Si_ is the effective length of the silicon wafer. The image was created using AutoCAD 2014 (simplified Chinese), http://win.xmjfg.com/pg/246.html.

MATLAB programs for the simulation calculation of the infrared spectra of s- and p-polarized light based on the MTR-IR method are shown in the Supporting Information. In Fig. 4, when s-polarized light enters the silicon chip at the Brewster incidence angle, *R*_*s*_ (reflection coefficient of s-polarized light on the surface of a silicon wafer) is equal to 0.71.

7 $${R}_{s}=0.71$$

8 $$\psi = 2\pi \sigma nb_{B}$$

9 $$T_{Si}^{s} = \frac{{(1 - R_{s} )^{2} + 4R_{s} \sin^{2} \delta }}{{[\exp (\frac{{\alpha b_{B} }}{2}) - R_{s} \exp ( - \frac{{\alpha b_{B} }}{2})]^{2} + 4R_{s} \sin^{2} (\psi + \delta )}}$$

10 $$R_{Si}^{s} = \frac{{R_{s} \left\{ {\left[ {\exp (\frac{{\alpha b_{B} }}{2}) - \exp ( - \frac{{\alpha b_{B} }}{2})} \right]^{2} + 4\sin^{2} \psi } \right\}}}{{[\exp (\frac{{\alpha b_{B} }}{2}) - R_{s} \exp ( - \frac{{\alpha b_{B} }}{2})]^{2} + 4R_{s} \sin^{2} (\psi + \delta )}}$$

where $\mathop T\nolimits_{Si}^{S}$ indicates the total transmittance of s-polarized light through the silicon wafer (including multiple transmissions within the silicon wafer). $\mathop R\nolimits_{Si}^{S}$ represents the total reflectance of s-polarized light on the surface of the silicon wafer (including multiple reflections within the silicon wafer). *δ* denotes the phase change caused by reflection in the absorbing medium. *n* is the refractive index of silicon. *σ* specifies the wavenumber, cm^−1^. *ψ* represents the phase change due to interfering multiple reflections on the boundaries of the sample. *α* is the absorption coefficient, cm^−1^.

### Simulation of thin-film optics for s-polarized light

In Fig. 4, when the transmission time *N* is equal to 1 or 2, the thin-film interference effect is considered according to thin-film optical theory.

11 $$\begin{gathered} T_{Si}^{s} + R_{Si}^{s} = \frac{{(1 - R_{s} )^{2} + 4R_{s} \sin^{2} \delta }}{{[\exp (\frac{{\alpha b_{B} }}{2}) - R_{s} \exp ( - \frac{{\alpha b_{B} }}{2})]^{2} + 4R_{s} \sin^{2} (\psi + \delta )}} \hfill \\ + \frac{{R_{s} \left\{ {\left[ {\exp (\frac{{\alpha b_{B} }}{2}) - \exp ( - \frac{{\alpha b_{B} }}{2})} \right]^{2} + 4\sin^{2} \psi } \right\}}}{{[\exp (\frac{{\alpha b_{B} }}{2}) - R_{s} \exp ( - \frac{{\alpha b_{B} }}{2})]^{2} + 4R_{s} \sin^{2} (\psi + \delta )}} \hfill \\ \end{gathered}$$

### Simulation of thick-film optics for s-polarized light

In Fig. 4, when *N* is greater than or equal to 3, the film thickness is calculated according to the thick-film optical principle, and the interference effect of the thin film is disregarded.

12 $$T_{Si}^{s} + R_{Si}^{s} = \frac{{(1 - R_{s} )^{2} \exp ( - \alpha b_{B} )}}{{1 - R_{s}^{2} \exp ( - 2\alpha b_{B} )}} + \frac{{R_{s} + (R_{s} - 2R_{s}^{2} )\exp ( - 2\alpha b_{B} )}}{{1 - R_{s}^{2} \exp ( - 2\alpha b_{B} )}}$$

**Figure 5:**
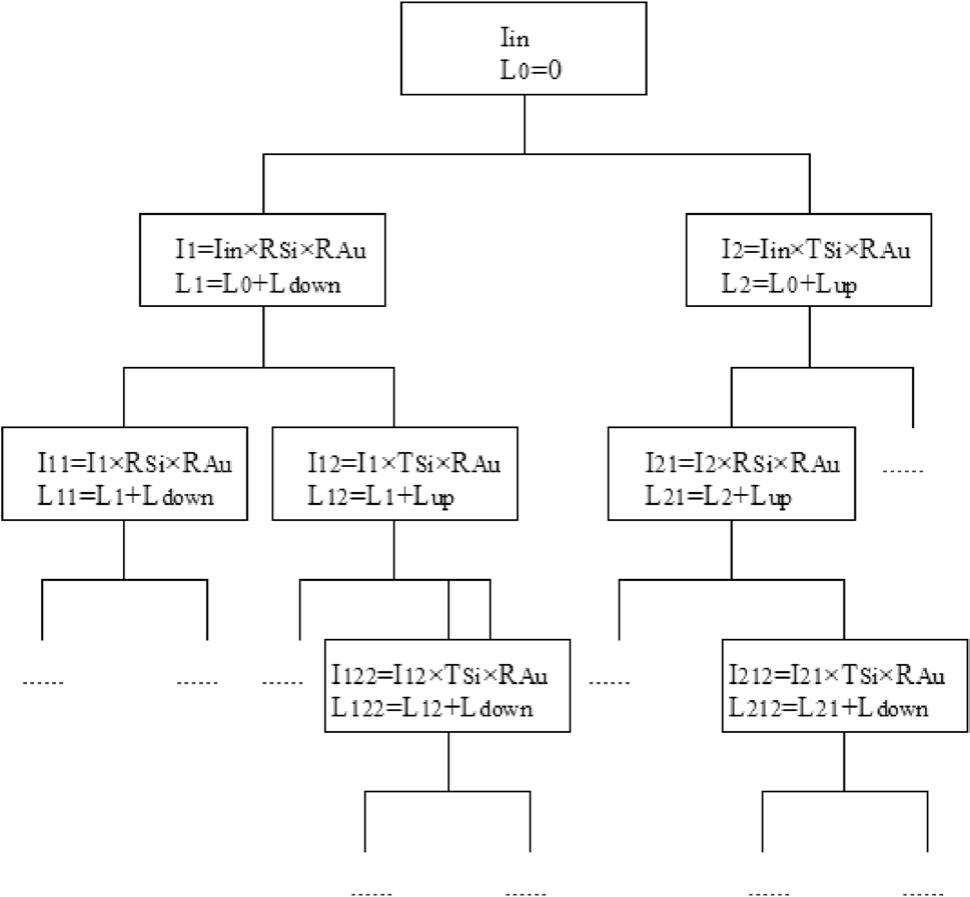
Flow chart of the calculation of the accumulative total intensity of output s-polarized light in the MTR configuration. I_in_ refers to the intensity of incident light. R_Si_ and T_Si_ denote the reflectance and transmittance of Si, respectively. The intensity in each branch is obtained by multiplication with R_Si_ and T_Si_ for reflection and transmission at the Si interface, respectively, and R_Au_ for reflection on the gold mirror. The subscripts indicate accumulative reflections and transmissions at the Si interface, where 1 and 2 represent one reflection and one transmission, respectively. The image was created using AutoCAD 2014 (simplified Chinese), http://win.xmjfg.com/pg/246.html.

Figure 5 exhibits the step-by-step calculations, in which the accumulative total intensity of the output s-polarized light can be obtained. When the incident light impinges on the silicon interface, it splits into reflection and transmission components and returns to the interface after reflection on gold mirrors. The split ray intensity is multiplied by T_Si_, R_Si_, or R_Au_ each time as light reaches these interfaces. After each step, the horizontal path length of the ray is increased by L_down_ or L_up_ (Fig. 5). The next calculation will be stopped when L is greater than L_Si_ or when the total intensity is less than 10^−4^. The sum of all terminal intensities of each branch in Fig. 5 corresponds to the accumulative total intensity of the output s-polarized light.

### Simulation and calculation of the infrared spectra of p-polarized light passing through a thin silicon wafer based on the MTR-IR method

**Figure 6:**
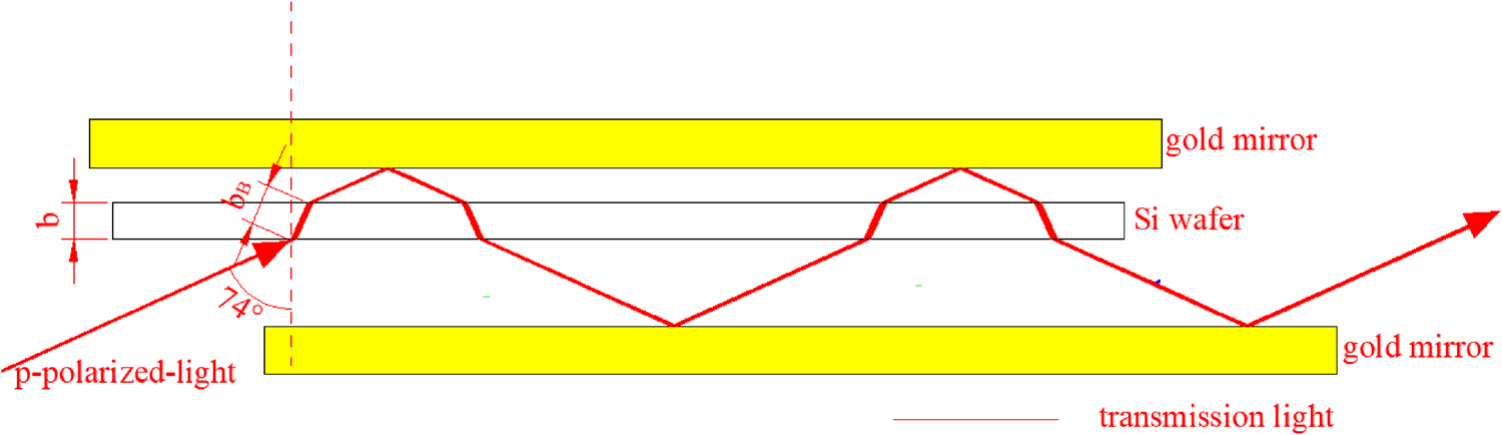
Light path chart of p-polarized light in the MTR setup.

In Fig. 6, when p-polarized light enters a silicon wafer at the Brewster incidence angle, *R*_*p*_ (the reflectivity of p-polarized light impinging on the silicon surface) is equal to 0. The image was created using AutoCAD 2014 (simplified Chinese), http://win.xmjfg.com/pg/246.html.

13 $$T_{Si}^{p} = \frac{{(1 - R_{p} )^{2} + 4R_{p} \sin^{2} \delta }}{{[\exp (\frac{{\alpha b_{B} }}{2}) - R_{p} \exp ( - \frac{{\alpha b_{B} }}{2})]^{2} + 4R_{p} \sin^{2} (\psi + \delta )}} \Rightarrow T_{Si}^{p} = \exp ( - \alpha b_{B} )$$

14 $$R_{Si}^{p} = \frac{{R_{p} \left\{ {[\exp (\frac{{\alpha b_{B} }}{2}) - \exp ( - \frac{{\alpha b_{B} }}{2})]^{2} + 4\sin^{2} \psi } \right\}}}{{[\exp (\frac{{\alpha b_{B} }}{2}) - R_{p} \exp ( - \frac{{\alpha b_{B} }}{2})]^{2} + 4R_{p} \sin^{2} (\psi + \delta )}} \Rightarrow R_{Si}^{p} = 0$$

$\mathop T\nolimits_{Si}^{p}$ represents the total transmittance of p-polarized light through the silicon wafer ($\mathop T\nolimits_{Si}^{p}$ is equal to 100% at the Brewster incidence angle). $\mathop R\nolimits_{Si}^{p}$ indicates the total reflectance of p-polarized light ($\mathop R\nolimits_{Si}^{p}$ is equal to 0 at the Brewster incidence angle).

### Simulation of thin-film optics for p-polarized light

In Fig. 6, when N is equal to 1 or 2, the interference effect of the thin film is considered.

15 $$T_{Si}^{p} + R_{Si}^{p} = \exp ( - \alpha b_{B} )$$

### Simulation of thick-film optics for p-polarized light

In Fig. 6, when N is greater than or equal to 3, thick-film theory is applied without considering the interference effect of the thin film.

16 $$T_{Si}^{p} = \frac{{(1 - R_{p} )^{2} \exp ( - \alpha b_{B} )}}{{1 - R_{p}^{2} \exp ( - 2\alpha b_{B} )}} \Rightarrow T_{Si}^{p} = \exp ( - \alpha b_{B} )$$

17 $$R_{Si}^{p} = \frac{{R_{p} + (R_{p} - 2R_{p}^{2} )\exp ( - 2\alpha b_{B} )}}{{1 - R_{p}^{2} \exp ( - 2\alpha b_{B} )}} \Rightarrow R_{Si}^{p} = 0$$

18 $$T_{Si}^{p} + R_{Si}^{p} = \exp ( - \alpha b_{B} )$$

The flow chart of the iterative calculation of p-polarized light is the same as that of s-polarized light (Fig. 5).

## Results and discussion

**Figure 7:**
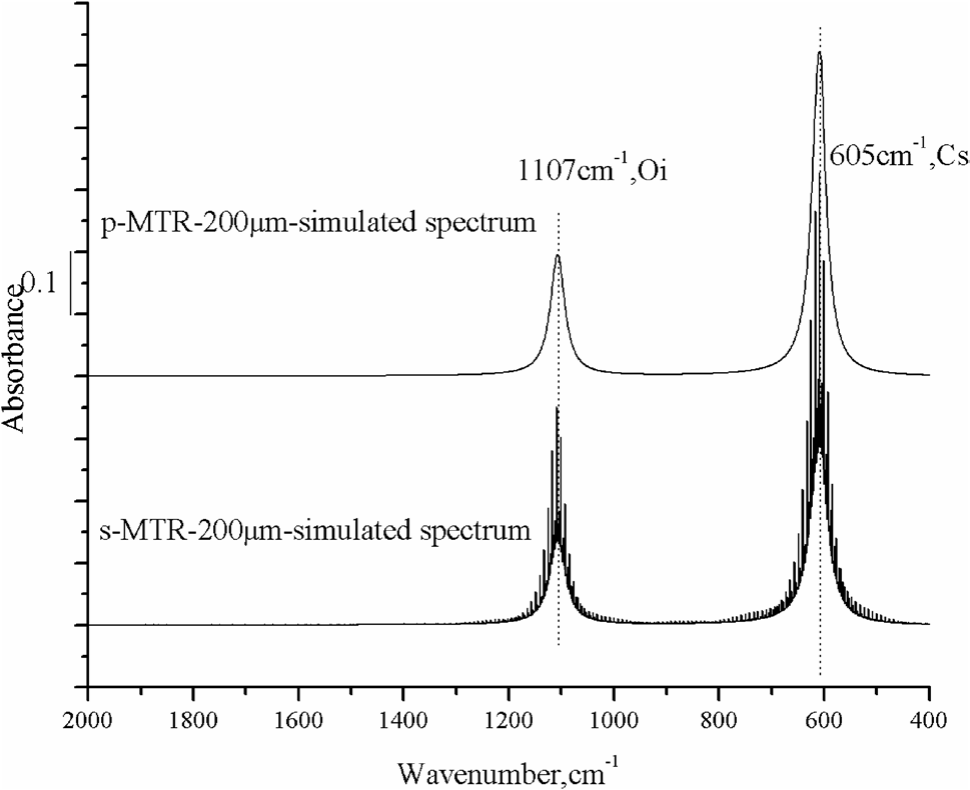
Simulated infrared spectra of substitutional carbon and interstitial oxygen in a thin silicon wafer 200 μm thick using p- and s-polarized light at the Brewster incidence angle. The parameters for calculation are b = 0.02 cm, incidence angle = 74°, N = 8, R_s_ = 0.71, and R_p_ = 0. The image was created using Origin 2017 (simplified Chinese), http://www.32r.com/soft/16769.html.

Figure 7 shows two simulated infrared spectra. The peak height of p-polarized light is higher than that of s-polarized light owing to the energy loss of reflection when s-polarized light reaches the silicon surface. Numerous interference fringes appear in the spectrum of s-polarized light, particularly at the absorption bands, due to thin-film interference from the silicon wafer with a 200 μm thickness.

**Figure 8:**
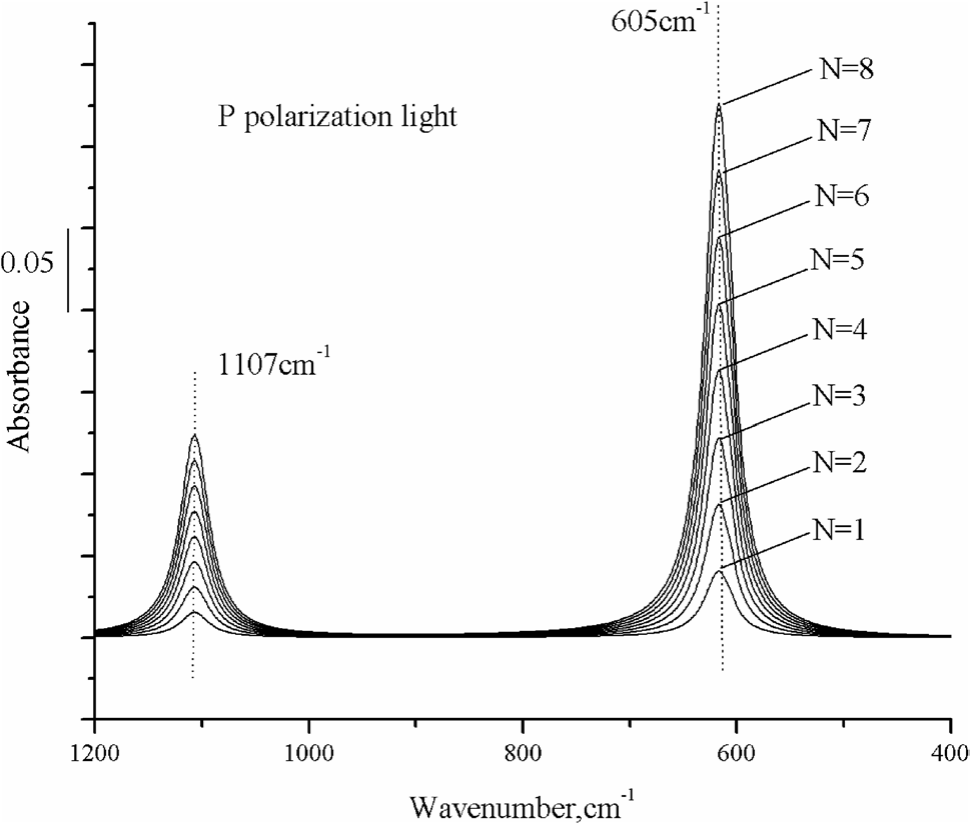
Simulated infrared spectra of substitutional carbon and interstitial oxygen in a thin silicon wafer using p-polarized light as a function of N. Parameters for calculation include N = 1 ~ 8, b = 0.02 cm, incidence angle = 74°, N = 8, and R_p_ = 0. The image was created using Origin 2017 (simplified Chinese), http://www.32r.com/soft/16769.html.

Figure 8 shows eight curves. The heights of the simulated absorption bands increase in proportion to N (from 1 to 8) when using p polarization. No interference fringes are observed, unlike with s-polarized light.

**Figure 9:**
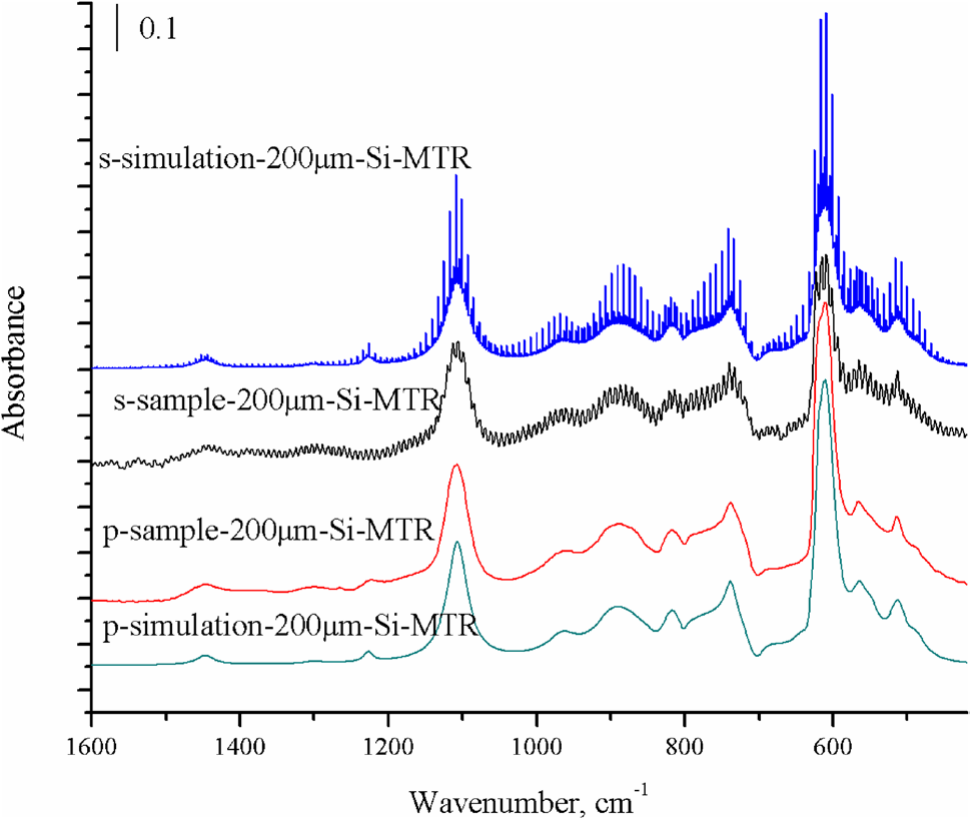
Comparison of sample and simulated infrared spectra of interstitial oxygen and substitutional carbon in a thin silicon wafer using s- and p-polarized light. The parameters for calculation are b = 0.02 cm, incidence angle = 74°, N = 8, R_s_ = 0.71, and R_p_ = 0. The image was created using Origin 2017 (simplified Chinese), http://www.32r.com/soft/16769.html.

Figure 9 shows four curves. There are no evident differences between the sample and simulated infrared spectra for p-polarized light, which are almost the same. However, the sample and simulated infrared spectra for s-polarized light show some differences, especially at the locations of absorption peaks. This might be caused by improper selection of certain parameters in the theoretical calculations. The peak heights in the sample and simulation infrared spectra for s polarization are lower than those for p polarization in Fig. 9.

### Analysis of the causes of the higher absorption peak for p-polarized light than that for s-polarized light

Higher absorption peaks are observed for p-polarized light than for s-polarized light for two possible reasons. (1) The vibration direction of p-polarized light is consistent with the main oscillation of Si–C and Si–O bonds. The vibration directions of s- and p-polarized light are perpendicular to each other. Thus, the principal vibration directions of Si–C and Si–O bonds are inconsistent with the s-polarized beam. (2) All of the p-polarized light passes totally through the silicon wafer at the Brewster incidence angle, and no reflection appears. Thus, all p-polarized rays reach the signal detector, and the maximum absorption peak is acquired. In contrast, 29% of the s-polarized light enters the silicon chip at the Brewster incidence angle because 71% of it is reflected. Therefore, less than 29% of the s-polarized light is obtained by the signal detector, and its minor absorption band is obtained.

## Conclusion

In this paper, the calculation of the content of interstitial oxygen and substitutional carbon in crystalline silicon was theoretically deduced based on the MTR-IR method, and the corresponding infrared spectra were simulated. By comparing the simulated spectra to the sample spectra, there are no differences between them, proving that the proposed principle model for calculation is appropriate. This theoretical simulation is not only applicable to the measurement of trace impurities in crystalline silicon but also suitable for quantitative characterization of trace impurities in other semiconductors by the MTR-IR method. This work might provide experimental operation and theoretical simulation support for research on the influence of the chromophore orientation and structure of organic molecules on the photoelectric conversion efficiency at room temperature and changes in interfacial characteristics, such as the limited dipole moment, charge separation, and photoelectric transmission at low temperature. It could be helpful for scientists in understanding the principles and mechanisms of solar cells and LED technologies, which is made easier by the proposed method.

## Supplementary Information

Supplementary Information
