# Supplementary material for: Simulation of infrared spectra of trace impurities in silicon wafers based on the multiple transmission–reflection infrared method
Source: Sci Rep. 2021 Jan 13;11:1254. doi: 10.1038/s41598-020-80883-0 (PMC7806787; doi:10.1038/s41598-020-80883-0)
Supplement: Supplementary file 1 — Supplementary Information [file 41598_2020_80883_MOESM1_ESM.docx]

Simulation of infrared spectra of trace impurities in silicon wafers based on the multiple transmission–reflection infrared method

Xiaobin Lu

(School of Chemistry and Chemical Engineering, Qiannan Normal University for Nationalities, Duyun 558000, Guizhou China)

Corresponding author: Xiaobin Lu (E-mail address: luxiaobin2007@163.com)

**1. MATLAB program for calculation of an integrating sphere**

%R&T and decreasing of calculation;

% air/Si/air=0/1/2

clear all;

fout=fopen('data.txt','w\n'); % or 'wt'

fprintf('k R T I\n');

for k = 1200:-1:1000;

n0=1;

n2=1;

d1=0.02;

theata0=74*pi/180;

theata1=16*pi/180;

theata2=74*pi/180;

beta=2*pi*k*d1;

c=3*10^10;

w=2.*pi.*k*c;

a1=10.3*10^34;

a2=2.55*10^35;

a3=0.5*10^33;

omiga1=2*pi*1107*c;

omiga2=2*pi*617*c;

omiga3=2*pi*605*c;

gama1=2*pi*c*33;

gama2=2*pi*c*32;

gama3=2*pi*c*6;

n1=3.42;

k1=a1*w./((omiga1^2-w.^2).^2+gama1^2*w.^2)+a2*w./((omiga2^2-w.^2).^2+gama2^2*w.^2)+a3*w./((omiga3^2-w.^2).^2+gama3^2*w.^2);

nz1=n1^2-k1^2-n0^2*sin(theata0)^2;

nz2=sqrt((n1^2-k1^2-n0^2*sin(theata0)^2)^2+4*n1^2*k1^2);

nz=sqrt(0.5*(nz1+nz2));

kz=sqrt(0.5*(-nz1+nz2));

r1=sqrt(((n0*cos(theata0)-nz)^2+kz^2)./((n0*cos(theata0)+nz)^2+kz^2));

t1=sqrt((2*n0*cos(theata0))^2./((n0*cos(theata0)+nz)^2+kz^2));

r2=sqrt(((n2*cos(theata2)-nz)^2+kz^2)./((n2*cos(theata2)+nz)^2+kz^2));

t2=sqrt(4*(nz^2+kz^2)./((n2*cos(theata2)+nz)^2+kz^2));

phai1r=atan(-2*kz*n0*cos(theata0)./(nz^2+kz^2-n0^2*cos(theata0)^2));

phai2r=atan(-2*kz*n2*cos(theata2)./(nz^2+kz^2-n2^2*cos(theata2)^2));

u=exp(-2*pi*k*d1*kz);

delta1=2*pi*k*d1*nz;

delta2=2*pi*k*d1*kz;

%A=(r1)^2+(r2)^2*u^4+2*(r1)*(r2)*u^2*cos(2*delta1+phai1r-phai2r);

%B=1+(r1)^2*(r2)^2*u^4+2*(r1)*(r2)*u^2*cos(2*delta1-phai1r-phai2r);

%R=A./B;

%C=(t1)^2*(t2)^2*u^2;

%D=1+(r1)^2*(r2)^2*u^4+2*(r1)*(r2)*u^2*cos(2*delta1-phai1r-phai2r);

%T=C./D;

%A=0.71+0.71*u^4+2*0.71*u^2*cos(2*delta1+phai1r-phai2r);

%B=1+0.71*0.71*u^4+2*0.71*u^2*cos(2*delta1-phai1r-phai2r);

%R=A./B+0.9;

%C=0.29*0.29*u^2;

%D=1+0.71*0.71*u^4+2*0.71*u^2*cos(2*delta1-phai1r-phai2r);

%T=C./D;

% nonpolarization

%A=0.3+0.3*u^4+2*0.3*u^2*cos(2*delta1+phai1r-phai2r);

%B=1+0.3*0.3*u^4+2*0.3*u^2*cos(2*delta1-phai1r-phai2r);

%R=A./B+0.9;

%C=0.7*0.7*u^2;

%D=1+0.3*0.3*u^4+2*0.3*u^2*cos(2*delta1-phai1r-phai2r);

%T=C./D;

A=(r1)^2+(r2)^2*u^4+2*(r1)*(r2)*u^2*cos(2*delta1+phai1r-phai2r);

B=1+(r1)^2*(r2)^2*u^4+2*(r1)*(r2)*u^2*cos(2*delta1-phai1r-phai2r);

R=A./B+0.2;

C=(t1)^2*(t2)^2*u^2;

D=1+(r1)^2*(r2)^2*u^4+2*(r1)*(r2)*u^2*cos(2*delta1-phai1r-phai2r);

T=C./D;

%A=(r1)^2*exp(2*beta*kz)+(r2)^2*exp(-2*beta*kz)+2*(r1)*(r2)*cos(phai1r-phai2r+2*nz*beta);

%B=exp(2*beta*kz)+(r1)^2*(r2)^2*exp(-2*beta*kz)+2*(r1)*(r2)*cos(phai1r+phai2r-2*nz*beta);

%R=A./B;

%C=(t1)^2*(t2)^2*exp(-2*beta*kz);

%D=1+(r1)^2*(r2)^2*exp(-4*beta*kz)+2*(r1)*(r2)*cos(phai1r+phai2r-2*nz*beta);

%T=C./D;

%A=abs(r1)^2*exp(2*beta*kz)+abs(r2)^2*exp(-2*beta*kz)+2*abs(r1)*abs(r2)*cos(phai1r-phai2r+2*nz*beta);

%B=exp(2*beta*kz)+abs(r1)^2*abs(r2)^2*exp(-2*beta*kz)+2*abs(r1)*abs(r2)*cos(phai1r+phai2r-2*nz*beta);

%R=A./B+0.5;

%C=abs(t1)^2*abs(t2)^2*exp(-2*beta*kz);

%D=1+abs(r1)^2*abs(r2)^2*exp(-4*beta*kz)+2*abs(r1)*abs(r2)*cos(phai1r+phai2r-2*nz*beta);

%T=C./D;

I=T+R;

fprintf(' %5.1f %4.6e %4.6e %4.6e\n',k, T, R, I);

fprintf(fout, ' %5.1f %4.6e %4.6e %4.6e\n',k, T, R, I);

end

fclose(fout);

A=load('data.txt');

v=A(:,1);

y1= A(:, 2);

y2= A(:, 3);

y3= A(:, 4);

plot(v,y1,'k');hold on;

plot(v,y2,'k');

plot(v,y3,'k');hold off;

%plot(v,y1,v,y2,v,y3,'r');

**2. MATLAB programs for simulation calculation of s- and p-polarized infrared spectra.**

% absorbance_matr_p

clear all;

fout=fopen('data.txt','w\n'); % or 'wt'

fprintf('k A_TR_s A_MTR_s A_MTR_p\n');

e_inf = 2.89;

S_0_x = 0.000002;

S_0_y = 0.0000002;

S_0_z = 0.000002;

k_0_x = 605;

k_0_y = 1107;

k_0_z = 610;

gama_0_x = 9;

gama_0_y = 10;

gama_0_z = 11;

angle_degree = 74;

d=2;

d_up=0.3;

R_s=0.71;

R_p=0;

d2=0.2;

R_Au=1;

L_Si_1=3;

L_Si_2=50;

for k = 400:1:4000;

n2_x = e_wavenumber_sub(k, e_inf, S_0_x, k_0_x, gama_0_x);

n2_z = e_wavenumber_sub(k, e_inf, S_0_z, k_0_z, gama_0_z);

n2_y = e_wavenumber_sub(k, e_inf, S_0_y, k_0_y, gama_0_y);

n= e_wavenumber_sub(k, e_inf, S_0_y, k_0_y, gama_0_y);

%n=n2_x+n2_y+n2_z;

A=2*pi*k*d2*real(n);

%n = e_wavenumber_sub(k, e_inf, S_0_x, k_0_x, gama_0_x);

%A=2*pi*k*d2*real(n);

B=pi;

%a=4*pi*k*imag(n2_x+n2_y+n2_z);

a=4*pi*k*imag(n);

%% thin film calculation

T_Si_p1=exp(-a*d2);

R_Si_p1=0 ;

%T_Si_p1=((1-R_p)^2+4*R_p*(sin(B))^2)./((exp(a*d2/2)-R_p*exp(-a*d2/2))^2+4*R_p*sin(B+A)^2);

%R_Si_p1=R_p*((exp(a*d2/2)-exp(-a*d2/2))^2+4*sin(A)^2)./((exp(a*d2/2)-R_p*exp(-a*d2/2))^2+4*R_p*sin(A+B)^2) ;

T_Si_s1=((1-R_s)^2+4*R_s*sin(B)^2)./((exp(a*d2/2)-R_s*exp(-a*d2/2))^2+4*R_s*sin(B+A)^2);

R_Si_s1=R_s*((exp(a*d2/2)-exp(-a*d2/2))^2+4*sin(A)^2)./((exp(a*d2/2)-R_s*exp(-a*d2/2))^2+4*R_s*sin(A+B)^2);

%T_Si_s=exp(-a*2)*(((1-R_s)^2-2*R_s*cos(2*B))./((1+R_s^2*exp(-a*d2/2))-2*R_s*exp(-a*d2)*cos(2*B+2*A)));

%R_Si_s=(R_s*((exp(a*d2/2)-exp(-a*d2/2))^2+4*sin(A)^2)./((exp(a*d2/2)-R_s*exp(-a*d2/2))^2+4*R_s*sin(A+B)^2));

I_output_s0=1;

%I_output_s1=inten_multi_R_T_1(angle_degree, L_Si_1, d, d_up, R_Si_s, T_Si_s, R_Au);

I_output_p0=1;

%I_output_p1=inten_multi_R_T_1(angle_degree, L_Si_1, d, d_up, R_Si_p, T_Si_p, R_Au);

A_GMBR_s = log10 (1./(R_Si_s1^2+T_Si_s1^2+T_Si_s1*R_Si_s1+T_Si_s1*R_Si_s1));

%%I_output_s1=R_Si_s^2+T_Si_s^2+2*R_Si_s*T_Si_s;

%%I_output_p1=R_Si_p^2+T_Si_p^2+2*R_Si_p*T_Si_p;

%I_output_s1=[R_Si_s1,T_Si_s1,0,0];

I_output_s1=[R_Si_s1^2,T_Si_s1^2,R_Si_s1*T_Si_s1,R_Si_s1*T_Si_s1];

% I_output_p1=[0,T_Si_p1,0,0];

I_output_p1=[R_Si_p1^2,T_Si_p1^2,R_Si_p1*T_Si_p1,R_Si_p1*T_Si_p1];

% A_MTR_s = log10(1./I_output_s_1);

%A_MTR_p = log10(1./I_output_p_1);

%%thick film calculation

T_Si_p=exp(-a*d2);

R_Si_p=0 ;

%T_Si_p=(1-R_p)^2*exp(-a*d2)./(1-R_p^2*exp(-2*a*d2));

%R_Si_p=R_p*((exp(a*d2/2)-exp(-a*d2/2))^2)./((exp(a*d2/2)-R_p*exp(-a*d2/2))^2) ;

T_Si_s=((1-R_s)^2*exp(-a*d2))./(1-R_s^2*exp(-2*a*d2));

R_Si_s=(R_s+R_s*((1-R_s)^2-R_s^2)*exp(-2*a*d2))./(1-R_s^2*exp(-2*a*d2));

%R_Si_s=R_s*((exp(a*d2/2)-exp(-a*d2/2))^2)./((exp(a*d2/2)-R_s*exp(-a*d2/2))^2);

%T_Si_s=(1-R_s)^2*exp(-a*d2)./(1-R_s^2*exp(-2*a*d2));

% R_Si_s=R_s*((exp(a*d2/2)-exp(-a*d2/2))^2)./((exp(a*d2/2)-R_s*exp(-a*d2/2))^2) ;

I_output_s0=1;

I_output_s=inten_multi_R_T_2(angle_degree, L_Si_2, d, d_up, R_Si_s, T_Si_s, R_Au,I_output_s1);

I_output_p0=1;

I_output_p=inten_multi_R_T_2(angle_degree, L_Si_2, d, d_up, R_Si_p, T_Si_p, R_Au,I_output_p1);

%A_GMBR_s = log10 (1./(T_Si_s^2));

A_MTR_s = log10(1./(I_output_s(1,1)+I_output_s(1,2)+I_output_s(1,3)+I_output_s(1,4)));

A_MTR_p = log10(1./(I_output_p(1,1)+I_output_p(1,2)+I_output_p(1,3)+I_output_p(1,4)));

% A_T_p_GMBR = log10 ((T_p_0.^2+R_p_0)./(T_p.^2+R_p));

fprintf(' %5.1f %4.6e %4.6e %4.6e\n',k, A_GMBR_s, A_MTR_s, A_MTR_p);

fprintf(fout, ' %5.1f %4.6e %4.6e %4.6e\n',k, A_GMBR_s,A_MTR_s, A_MTR_p);

end,

fclose(fout);

A=load('data.txt');

v=A(:,1);

y1= A(:, 2);

y2= A(:, 3);

y3= A(:, 4);

%plot(v,y3,'b');

plot(v,y2,'r',v,y3,'b');

******************************************************************************

%e_wavenumber_sub

function n = e_wavenumber_sub(k, e_inf, S_0, k_0, gama_0)

%e_k = e_inf + S_0.*k_0^2/(k_0^2-k^2-i*gama_0*k);

%n = sqrt(e_k);

c=3*10^10;

w=2.*pi.*k*c;

a1=0.64*17.3*10^34.2;

a2=0.41*78*10^32.2;

a3=0.51*285*10^33.2;

a4=0.74*0.963*10^34.2;

a5=0.74*0.233*10^34.2;

a6=0.74*0.253*10^34.2;

a7=0.74*4.9*10^34.2;

a14=0.74*5*10^34.2;

a8=2.74*5*10^34.2;

a9=2.74*0.39*10^34.2;

a13=0.74*141*10^34.2;

a10=0.74*1.8*10^34.2;

a11=0.74*4*10^34.2;

a12=0.74*4*10^34.2;

a15=2*10^34.2;

a16=0.4*10^34.2;

a17=0.12*15*10^33.2;

a18=2.94*0.19*10^34.2;

a19=2.14*0.07*10^34.2;

a20=0.74*3*10^34.2;

a21=0.74*2*10^34.2;

a22=4.8*10^34.2;

omiga1=2*pi*1107*c;

omiga2=2*pi*620*c;

omiga3=2*pi*609*c;

omiga4=2*pi*1447*c;

omiga5=2*pi*1301*c;

omiga6=2*pi*1227*c;

omiga7=2*pi*966*c;

omiga14=2*pi*902*c;

omiga8=2*pi*880*c;

omiga9=2*pi*818*c;

omiga13=2*pi*753*c;

omiga10=2*pi*738*c;

omiga11=2*pi*565*c;

omiga12=2*pi*513*c;

omiga15=2*pi*705*c;

omiga16=2*pi*635*c;

omiga17=2*pi*615*c;

omiga18=2*pi*838*c;

omiga19=2*pi*802*c;

omiga20=2*pi*548*c;

omiga21=2*pi*487*c;

omiga22=2*pi*660*c;

gama1=2*pi*c*33;

gama2=2*pi*c*7;

gama3=2*pi*c*26;

gama4=2*pi*c*30;

gama5=2*pi*c*30;

gama6=2*pi*c*15;

gama7=2*pi*c*48;

gama14=2*pi*c*48;

gama8=2*pi*c*73;

gama9=2*pi*c*25;

gama13=2*pi*c*160;

gama10=2*pi*c*20;

gama11=2*pi*c*28;

gama12=2*pi*c*27;

gama15=2*pi*c*28;

gama16=2*pi*c*14;

gama17=2*pi*c*8;

gama18=2*pi*c*25;

gama19=2*pi*c*12;

gama20=2*pi*c*30;

gama21=2*pi*c*31;

gama22=2*pi*c*74;

ep2=-a22*w./((omiga22^2-w.^2).^2+gama22^2*w.^2)+a20*w./((omiga20^2-w.^2).^2+gama20^2*w.^2)+a21*w./((omiga21^2-w.^2).^2+gama21^2*w.^2)-a19*w./((omiga19^2-w.^2).^2+gama19^2*w.^2)-a18*w./((omiga18^2-w.^2).^2+gama18^2*w.^2)-a16*w./((omiga16^2-w.^2).^2+gama16^2*w.^2)+a17*w./((omiga17^2-w.^2).^2+gama17^2*w.^2)-a15*w./((omiga15^2-w.^2).^2+gama15^2*w.^2)+a14*w./((omiga14^2-w.^2).^2+gama14^2*w.^2)+a13*w./((omiga13^2-w.^2).^2+gama13^2*w.^2)+a1*w./((omiga1^2-w.^2).^2+gama1^2*w.^2)+a2*w./((omiga2^2-w.^2).^2+gama2^2*w.^2)+a3*w./((omiga3^2-w.^2).^2+gama3^2*w.^2)+a4*w./((omiga4^2-w.^2).^2+gama4^2*w.^2)+a5*w./((omiga5^2-w.^2).^2+gama5^2*w.^2)+a6*w./((omiga6^2-w.^2).^2+gama6^2*w.^2)+a7*w./((omiga7^2-w.^2).^2+gama7^2*w.^2)+a8*w./((omiga8^2-w.^2).^2+gama8^2*w.^2)+a9*w./((omiga9^2-w.^2).^2+gama9^2*w.^2)+a10*w./((omiga10^2-w.^2).^2+gama10^2*w.^2)+a11*w./((omiga11^2-w.^2).^2+gama11^2*w.^2)+a12*w./((omiga12^2-w.^2).^2+gama12^2*w.^2);

n=11.56+ep2*i;

******************************************************************************

%inten_multi_R_T_1

function I_output=inten_multi_R_T(angle_degree, L_Si, d, d_up, R_Si, T_Si, R_Au)

% calculation of the output intensity of the multiple

% transmission_reflection

% angle; incident angle

% L_Si; length of silcon wafer

% d; distance between two sample mirror

% d_up; distance between silicon wafer and the up_mirror

% (light come from the other side of silicon wafer)

% R_Si reflectance of double-polish-silicon wafer

% T_Si transmitance of double-polish-silicon wafer

angle_degree = 74;

d=2;

d_up=0.3;

d_Si=0.2;

angle = angle_degree*pi/180;

R_Au=1;

L_Si=3;

% thickness of silicon wafer

d_down = d-d_Si-d_up; %distance between silicon wafer and the down_mirror

angle_Si = asin(1/3.42*sin(angle));

L_in_Si = d_Si*tan(angle_Si);

L_up = 2*(d_up*tan(angle) + L_in_Si); % the length light passed on up silcion wafer

L_down = 2*(d_down*tan(angle)); % the length light passed on down silcion wafer

multi(1,1) = 1;

multi(1,2) = 1;

multi(1,3) = 0;

I_output = 0;

Line = 1; % the root number

while isempty(multi)==0

for I = 1:Line

% reflection

inten_R(I,1) = multi(I,1).*R_Si.*R_Au;

inten_R(I,2) = multi(I,2).*1;

if inten_R(I,1)<1E-10

inten_R(I,1:3) = 0;

end

if inten_R(I,2)==1

inten_R(I,3) = multi(I,3)+L_down; % if the inten_R(I,3)=1, add the L_down to length

elseif inten_R(I,2)==-1

inten_R(I,3) = multi(I,3)+L_up; % if the inten_R(I,3)=-1, add the L_up to length

end

% transmission

inten_T(I,1) = multi(I,1).*T_Si.*R_Au;

inten_T(I,2) = multi(I,2).*(-1);

if inten_T(I,1)<1E-10

inten_T(I,1:3) = 0;

end

if inten_T(I,2)==1

inten_T(I,3) = multi(I,3)+L_down; % if the inten_T(I,3)=1, add the L_down to length

elseif inten_T(I,2)==-1

inten_T(I,3) = multi(I,3)+L_up; % if the inten_T(I,3)=-1, add the L_up to length

end

% save reflection light

if inten_R(I,3)<=L_Si + 1E-10

multi(I,:) = inten_R(I,:);

else % light go out the two mirrors and save the intensity

I_output = I_output+inten_R(I,1);

multi(I,:) = 0;

end

% save transmission light

if inten_T(I,3)<=L_Si + 1E-10

multi(I+Line,:) = inten_T(I,:);

else % light go out the two mirrors and save the intensity

I_output = I_output+inten_T(I,1);

multi(I+Line,:) = 0;

end

end

inten_R = 0;

inten_T = 0;

Q=1;

for P = 1:(Line+I)

if multi(Q,:) == 0

multi(Q,:) = []; % delete the "0" element

Q = Q-1; % change array size

end

Q = Q+1;

end

[Line,Colm] = size(multi);

End

*******************************************************************************

%inten_multi_R_T_2

function I_output=inten_multi_R_T(angle_degree, L_Si, d, d_up, R_Si, T_Si, R_Au,I_output_s1)

% calculation of the output intensity of the multiple

% transmission_reflection

% angle; incident angle

% L_Si; length of silcon wafer

% d; distance between two sample mirror

% d_up; distance between silicon wafer and the up_mirror

% (light come from the other side of silicon wafer)

% R_Si reflectance of double-polish-silicon wafer

% T_Si transmitance of double-polish-silicon wafer

angle_degree = 74;

d=2;

d_up=0.3;

d_Si=0.2;

angle = angle_degree*pi/180;

R_Au=1;

L_Si=50;

% thickness of silicon wafer

d_down = d-d_Si-d_up; %distance between silicon wafer and the down_mirror

angle_Si = asin(1/3.42*sin(angle));

L_in_Si = d_Si*tan(angle_Si);

L_up = 2*(d_up*tan(angle) + L_in_Si); % the length light passed on up silcion wafer

L_down = 2*(d_down*tan(angle)); % the length light passed on down silcion wafer

multi(1,1) = I_output_s1(1,1);

multi(1,2) = 1;

multi(1,3) = 0;

I_output(1,1) = 0;

Line = 1; % the root number

while isempty(multi)==0

for I = 1:Line

% reflection

inten_R(I,1) = multi(I,1).*R_Si.*R_Au;

inten_R(I,2) = multi(I,2).*1;

if inten_R(I,1)<1E-10

inten_R(I,1:3) = 0;

end

if inten_R(I,2)==1

inten_R(I,3) = multi(I,3)+L_down; % if the inten_R(I,3)=1, add the L_down to length

elseif inten_R(I,2)==-1

inten_R(I,3) = multi(I,3)+L_up; % if the inten_R(I,3)=-1, add the L_up to length

end

% transmission

inten_T(I,1) = multi(I,1).*T_Si.*R_Au;

inten_T(I,2) = multi(I,2).*(-1);

if inten_T(I,1)<1E-10

inten_T(I,1:3) = 0;

end

if inten_T(I,2)==1

inten_T(I,3) = multi(I,3)+L_down; % if the inten_T(I,3)=1, add the L_down to length

elseif inten_T(I,2)==-1

inten_T(I,3) = multi(I,3)+L_up; % if the inten_T(I,3)=-1, add the L_up to length

end

% save reflection light

if inten_R(I,3)<=L_Si + 1E-10

multi(I,:) = inten_R(I,:);

else % light go out the two mirrors and save the intensity

I_output(1,1) = I_output(1,1)+inten_R(I,1);

multi(I,:) = 0;

end

% save transmission light

if inten_T(I,3)<=L_Si + 1E-10

multi(I+Line,:) = inten_T(I,:);

else % light go out the two mirrors and save the intensity

I_output(1,1) = I_output(1,1)+inten_T(I,1);

multi(I+Line,:) = 0;

end

end

inten_R = 0;

inten_T = 0;

Q=1;

for P = 1:(Line+I)

if multi(Q,:) == 0

multi(Q,:) = []; % delete the "0" element

Q = Q-1; % change array size

end

Q = Q+1;

end

[Line,Colm] = size(multi);

end

multi(1,1) = I_output_s1(1,2);

multi(1,2) = 1;

multi(1,3) = 0;

I_output(1,2) = 0;

Line = 1; % the root number

while isempty(multi)==0

for I = 1:Line

% reflection

inten_R(I,1) = multi(I,1).*R_Si.*R_Au;

inten_R(I,2) = multi(I,2).*1;

if inten_R(I,1)<1E-10

inten_R(I,1:3) = 0;

end

if inten_R(I,2)==1

inten_R(I,3) = multi(I,3)+L_down; % if the inten_R(I,3)=1, add the L_down to length

elseif inten_R(I,2)==-1

inten_R(I,3) = multi(I,3)+L_up; % if the inten_R(I,3)=-1, add the L_up to length

end

% transmission

inten_T(I,1) = multi(I,1).*T_Si.*R_Au;

inten_T(I,2) = multi(I,2).*(-1);

if inten_T(I,1)<1E-10

inten_T(I,1:3) = 0;

end

if inten_T(I,2)==1

inten_T(I,3) = multi(I,3)+L_down; % if the inten_T(I,3)=1, add the L_down to length

elseif inten_T(I,2)==-1

inten_T(I,3) = multi(I,3)+L_up; % if the inten_T(I,3)=-1, add the L_up to length

end

% save reflection light

if inten_R(I,3)<=L_Si + 1E-10

multi(I,:) = inten_R(I,:);

else % light go out the two mirrors and save the intensity

I_output(1,2) = I_output(1,2)+inten_R(I,1);

multi(I,:) = 0;

end

% save transmission light

if inten_T(I,3)<=L_Si + 1E-10

multi(I+Line,:) = inten_T(I,:);

else % light go out the two mirrors and save the intensity

I_output(1,2) = I_output(1,2)+inten_T(I,1);

multi(I+Line,:) = 0;

end

end

inten_R = 0;

inten_T = 0;

Q=1;

for P = 1:(Line+I)

if multi(Q,:) == 0

multi(Q,:) = []; % delete the "0" element

Q = Q-1; % change array size

end

Q = Q+1;

end

[Line,Colm] = size(multi);

end

multi(1,1) = I_output_s1(1,3);

multi(1,2) = 1;

multi(1,3) = 0;

I_output(1,3) = 0;

Line = 1; % the root number

while isempty(multi)==0

for I = 1:Line

% reflection

inten_R(I,1) = multi(I,1).*R_Si.*R_Au;

inten_R(I,2) = multi(I,2).*1;

if inten_R(I,1)<1E-10

inten_R(I,1:3) = 0;

end

if inten_R(I,2)==1

inten_R(I,3) = multi(I,3)+L_down; % if the inten_R(I,3)=1, add the L_down to length

elseif inten_R(I,2)==-1

inten_R(I,3) = multi(I,3)+L_up; % if the inten_R(I,3)=-1, add the L_up to length

end

% transmission

inten_T(I,1) = multi(I,1).*T_Si.*R_Au;

inten_T(I,2) = multi(I,2).*(-1);

if inten_T(I,1)<1E-10

inten_T(I,1:3) = 0;

end

if inten_T(I,2)==1

inten_T(I,3) = multi(I,3)+L_down; % if the inten_T(I,3)=1, add the L_down to length

elseif inten_T(I,2)==-1

inten_T(I,3) = multi(I,3)+L_up; % if the inten_T(I,3)=-1, add the L_up to length

end

% save reflection light

if inten_R(I,3)<=L_Si + 1E-10

multi(I,:) = inten_R(I,:);

else % light go out the two mirrors and save the intensity

I_output(1,3) = I_output(1,3)+inten_R(I,1);

multi(I,:) = 0;

end

% save transmission light

if inten_T(I,3)<=L_Si + 1E-10

multi(I+Line,:) = inten_T(I,:);

else % light go out the two mirrors and save the intensity

I_output(1,3) = I_output(1,3)+inten_T(I,1);

multi(I+Line,:) = 0;

end

end

inten_R = 0;

inten_T = 0;

Q=1;

for P = 1:(Line+I)

if multi(Q,:) == 0

multi(Q,:) = []; % delete the "0" element

Q = Q-1; % change array size

end

Q = Q+1;

end

[Line,Colm] = size(multi);

end

multi(1,1) = I_output_s1(1,4);

multi(1,2) = 1;

multi(1,3) = 0;

I_output(1,4) = 0;

Line = 1; % the root number

while isempty(multi)==0

for I = 1:Line

% reflection

inten_R(I,1) = multi(I,1).*R_Si.*R_Au;

inten_R(I,2) = multi(I,2).*1;

if inten_R(I,1)<1E-10

inten_R(I,1:3) = 0;

end

if inten_R(I,2)==1

inten_R(I,3) = multi(I,3)+L_down; % if the inten_R(I,3)=1, add the L_down to length

elseif inten_R(I,2)==-1

inten_R(I,3) = multi(I,3)+L_up; % if the inten_R(I,3)=-1, add the L_up to length

end

% transmission

inten_T(I,1) = multi(I,1).*T_Si.*R_Au;

inten_T(I,2) = multi(I,2).*(-1);

if inten_T(I,1)<1E-10

inten_T(I,1:3) = 0;

end

if inten_T(I,2)==1

inten_T(I,3) = multi(I,3)+L_down; % if the inten_T(I,3)=1, add the L_down to length

elseif inten_T(I,2)==-1

inten_T(I,3) = multi(I,3)+L_up; % if the inten_T(I,3)=-1, add the L_up to length

end

% save reflection light

if inten_R(I,3)<=L_Si + 1E-10

multi(I,:) = inten_R(I,:);

else % light go out the two mirrors and save the intensity

I_output(1,4) = I_output(1,4)+inten_R(I,1);

multi(I,:) = 0;

end

% save transmission light

if inten_T(I,3)<=L_Si + 1E-10

multi(I+Line,:) = inten_T(I,:);

else % light go out the two mirrors and save the intensity

I_output(1,4) = I_output(1,4)+inten_T(I,1);

multi(I+Line,:) = 0;

end

end

inten_R = 0;

inten_T = 0;

Q=1;

for P = 1:(Line+I)

if multi(Q,:) == 0

multi(Q,:) = []; % delete the "0" element

Q = Q-1; % change array size

end

Q = Q+1;

end

[Line,Colm] = size(multi);

end
